# Editorial: Advances and challenges in AI-driven visual intelligence: bridging theory and practice

**DOI:** 10.3389/frai.2025.1740331

**Published:** 2025-11-27

**Authors:** Bo Huang, Dawei Zhang, Qiao Liu

**Affiliations:** 1 College of Optoelectronic Engineering, Chongqing University (CQU), Chongqing, China; 2 Department of Computer Science and Technology, Zhejiang Normal University, Jinhua, China; 3 National Center for Applied Mathematics, Chongqing Normal University, Chongqing, China

**Keywords:** AI-driven visual intelligence, adversarial attacks, semi-supervised learning, 3D reconstruction, non-invasive diagnostic

Visual intelligence has become a fundamental research area in artificial intelligence, driven by rapid advances in deep learning and increasing demands from practical applications. This Research Topic brings together cutting-edge research that addresses both the remarkable progress and the persistent challenges in implementing AI-driven visual intelligence systems in real-world applications. The overview of this Research Topic is shown in Figure 1. The six articles published in this collection exemplify the breadth and depth of current research, spanning from fundamental security concerns to practical applications across diverse fields.

**Figure 1:**
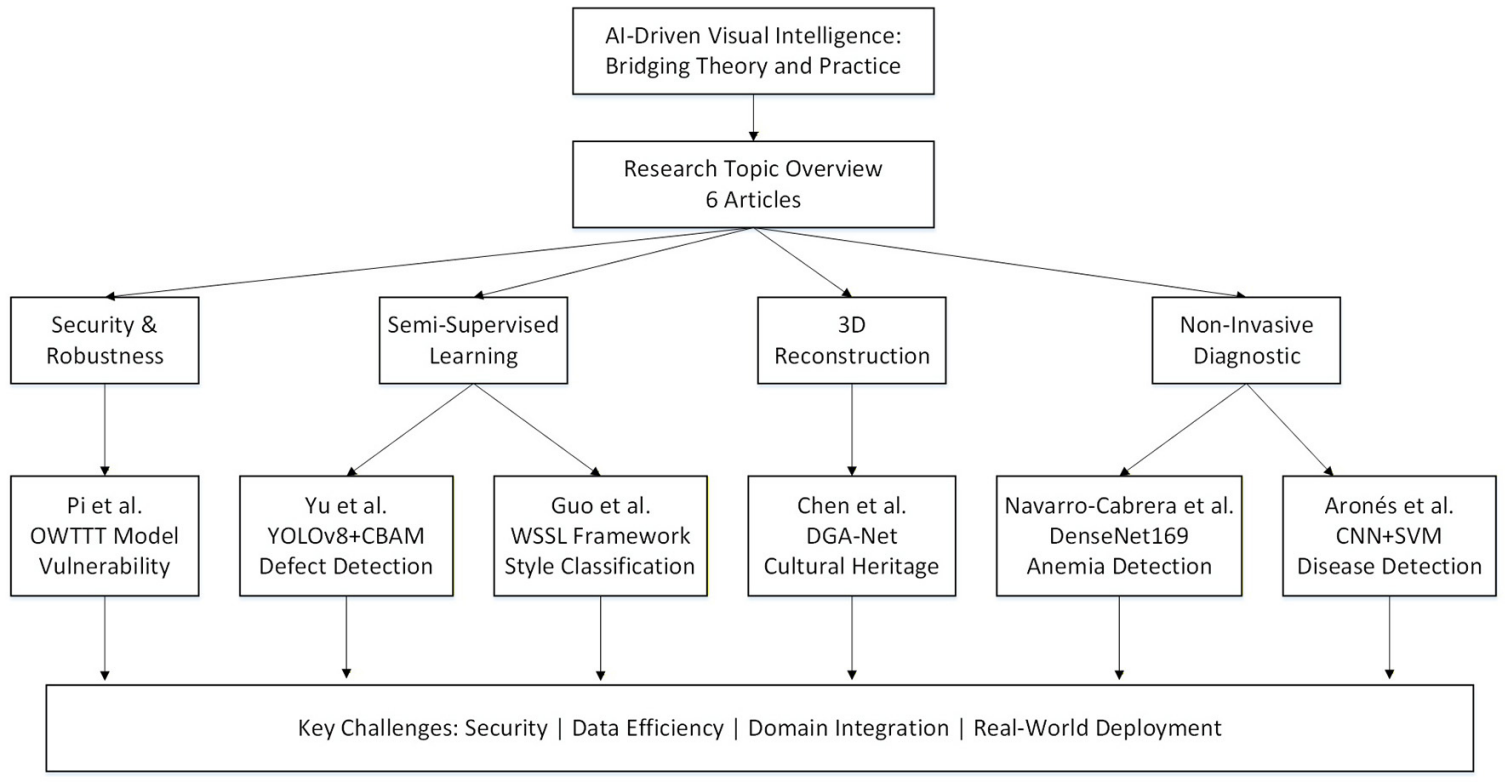
Overview of this Research Topic.

## Robustness against adversarial attacks

As AI systems increasingly operate in dynamic, open-world scenarios, their vulnerability to adversarial manipulation presents significant concerns. Pi et al. investigate test-time poisoning attacks targeting open-world test-time training (OWTTT) models. Their findings reveal that these models can be compromised with merely 100 queries using single-step query-based attack strategies. More critically, the affected models show limited recovery capability even when subsequently exposed to normal samples. This work serves as a crucial reminder that as we pursue adaptive AI systems, we must simultaneously address their security vulnerabilities to ensure safe deployment in critical applications.

## Semi-supervised learning frameworks

The scarcity of labeled training data remains a persistent bottleneck in visual intelligence applications. Two articles tackle this limitation through innovative semi-supervised approaches. Yu et al. propose an enhanced YOLOv8 framework that embeds the CBAM attention mechanism in high-level networks while incorporating Mean Teacher semi-supervised learning to address data labeling challenges. Their method achieves robust detection of micron-scale defects in industrial polymer films. Guo et al. introduce a weight-aware semi-supervised self-ensembling framework (WSSL) for interior decoration style classification. By employing an adaptive weighting module based on truncated Gaussian functions, their approach selectively leverages reliable unlabeled data while mitigating confirmation bias from unreliable pseudo-labels. Both works demonstrate that semi-supervised learning can substantially reduce annotation costs without compromising performance.

## 3D reconstruction and cultural heritage

The preservation of cultural landscapes presents unique challenges that require accurate 3D reconstruction capabilities. Chen et al. develop DGA-Net, which combines deep feature extraction with graph structure representation for reconstructing historical garden landscapes. The architecture incorporates attention mechanisms to emphasize ecologically significant features, enabling more accurate restoration planning and cultural heritage protection. This work exemplifies how AI-driven visual intelligence can contribute to environmental conservation and the preservation of cultural heritage sites.

## Non-invasive diagnostic applications

Two articles demonstrate the potential of visual intelligence for accessible healthcare and agricultural monitoring. Navarro-Cabrera et al. apply DenseNet169 for detecting iron deficiency anemia in university students through fingernail image analysis. Their approach achieves 71.08% accuracy with 74.09% AUC, offering a cost-effective alternative to conventional blood tests, particularly relevant for developing regions. Aronés et al. present App2, a mobile application combining CNN and SVM architectures for apple leaf disease detection. The system achieves 95% accuracy on clear images and maintains 80% performance under real-field conditions after model adaptation. Notably, their inclusion of validation filters to verify leaf presence reduces false detections, demonstrating attention to practical deployment considerations. Both works highlight how AI-based visual intelligence can address accessibility gaps in healthcare and agriculture.

## Open challenges and future directions

The collected works reveal several persistent challenges in visual intelligence research. First, the security of adaptive systems requires greater attention, particularly as models operate in adversarial environments. The difficulty in recovering from poisoning attacks suggests that defensive mechanisms must be incorporated at the architectural level. Second, efficient learning from limited labeled data continues to demand innovation, with semi-supervised and self-supervised methods showing promise but requiring careful handling of pseudo-label reliability. Third, the integration of domain knowledge with deep learning architectures remains an underexplored avenue that could enhance both performance and interpretability. Finally, the gap between laboratory performance and real-world deployment—exemplified by the accuracy drop in field conditions—indicates that robustness to environmental variations needs greater emphasis during model development.

The progress documented in this Research Topic demonstrates that effective visual intelligence systems require more than algorithmic sophistication. Success depends equally on addressing practical constraints including computational efficiency, data availability, security vulnerabilities, and deployment accessibility. As the field advances, bridging the gap between theoretical capabilities and practical requirements will necessitate continued collaboration across disciplines and careful consideration of real-world operating conditions.

